# Prediction of Metastasis and Recurrence in Colorectal Cancer Based on Gene Expression Analysis: Ready for the Clinic?

**DOI:** 10.3390/cancers3032858

**Published:** 2011-07-07

**Authors:** Masaki Shibayama, Matthias Maak, Ulrich Nitsche, Kengo Gotoh, Robert Rosenberg, Klaus-Peter Janssen

**Affiliations:** 1 Sysmex Corporation, Central Research Laboratories, Kobe 651-2271, Japan; E-Mail: Shibayama.Masaki@sysmex.co.jp (M.S.); Gotoh.Kengo@sysmex.co.jp (K.G.); 2 Chirurgische Klinik, Klinikum Rechts der Isar der TUM, München 81657, Germany; E-Mails: maak@chir.med.tu-muenchen.de (M.M.); nitsche@chir.med.tu-muenchen.de (U.N.); rosenberg@chir.med.tu-muenchen.de (R.R.)

**Keywords:** colorectal cancer, transcriptome, metastasis, prognosis, therapy prediction

## Abstract

Cancers of the colon and rectum, which rank among the most frequent human tumors, are currently treated by surgical resection in locally restricted tumor stages. However, disease recurrence and formation of local and distant metastasis frequently occur even in cases with successful curative resection of the primary tumor (R0). Recent technological advances in molecular diagnostic analysis have led to a wealth of knowledge about the changes in gene transcription in all stages of colorectal tumors. Differential gene expression, or transcriptome analysis, has been proposed by many groups to predict disease recurrence, clinical outcome, and also response to therapy, in addition to the well-established clinico-pathological factors. However, the clinical usability of gene expression profiling as a reliable and robust prognostic tool that allows evidence-based clinical decisions is currently under debate. In this review, we will discuss the most recent data on the prognostic significance and potential clinical application of genome wide expression analysis in colorectal cancer.

## 1. Introduction

Colorectal cancer (CRC) is one of the most common cancers and the second leading cause of cancer-related death worldwide [1,2]. The progression of colorectal cancer is well documented in histopathological terms, and the molecular genetic changes associated with the so-called “adenoma-carcinoma” tumor progression sequence have been studied extensively over the last decades. Several types of staging systems have been developed for colorectal cancer, such as the Dukes' system, the modified Astler-Coller staging system and the TNM system introduced by the American Joint Committee on Cancer (AJCC) and the International Union Against Cancer (UICC) [3-6]. These staging systems rely on the size and extent of the primary tumor, on the metastatic spread to lymph nodes and distant organ sites, and on lymphatic and vascular invasion (TNM system). Metastasis formation is the major cause of death in patients with colorectal cancer, and depending on tumor stage, liver metastases occur in 20% to 70% of patients, and lung metastases in 10% to 20% of cases. For patients with locally restricted colon tumors without lymph node metastasis, surgical tumor resection is the current standard therapy. Adjuvant chemotherapy (radio-chemotherapy in the case of rectal cancer) is generally only recommended for more advanced tumor stages. However, all current staging systems have their shortcomings and limitations. To state an example of high clinical relevance: it is not possible to prospectively identify the high-risk group of 20–30% of patients with locally restricted stage II (UICC) colon cancer that will suffer from disease recurrence. This group of patients may actually benefit from adjuvant therapy in addition to surgical treatment, even though chemotherapy is not recommended according to current guidelines [7].

Recently, increasing knowledge of the molecular etiology of tumor progression from adenoma to adenocarcinoma of the colon has facilitated the identification of a number of prognostic and predictive biomarker candidates. A sequential process of epigenetic alterations and gene mutations is widely believed to drive this process, and the acquisition of these mutations is facilitated by the loss of genomic stability, which occurs in two major forms: as chromosomal instability (CIN), and, less frequently, as microsatellite instability (MSI). A relatively small number of signaling pathways seems to drive the progression of colorectal cancer, since aberrant activation of the canonical WNT-pathway, mutations in the oncogene KRAS and the tumor suppressor TP53 can be found in the majority of all cases of sporadic colorectal cancer. However, next to these essential “driver” mutations, a multitude of “passenger” mutations or epigenetic alterations are likely to occur, contributing to heterogeneity. The clinical usefulness of detecting oncogenic KRAS mutations, mutations and loss of p53 function, or loss of heterozygosity of chromosome18q, microsatellite and chromosomal instability status have been intensively investigated in the light of their prognostic capacity. Despite these enormous efforts aimed at finding molecular markers for individualized medicine, the ASCO 2006 guidelines for use of tumor markers in colorectal cancer did not conclude that there was sufficient data to support the use of any of these markers [8]. However, the use of KRAS mutations to predict the efficacy of a therapy directed against the EGFR (epidermal growth factor receptor) has been well documented [9].

DNA microarray technology, the so-called “gene chips”, is one of the comprehensive or “omics” approaches which allows the concomitant profiling of the expression of thousands of genes, reflecting the global transcription activity at one given point in time. This approach has been widely used in medical oncology, with a good potential to provide a detailed and comprehensive view of gene expression changes involved in tumor initiation and progression. Moreover, this method holds the promise to determine the sensitivity towards classical cytotoxic chemo/radio-therapies, or the new class of “biological” like the anti-EGFR therapy, and allows an insight into the underlying resistance mechanisms. However, even though much has been learned about cancer biology from transcriptome profiling of normal colon mucosa as compared to carcinoma tissue, the clinical application of microarray technology is currently very limited in gastrointestinal oncology. If the hypothesis is correct that a limited number of critical “driver” pathways determine clinical outcome in most cancers, the results from transcriptome studies should identify highly similar subsets of transcripts. As an example, the WNT-pathway is activated by mutations in the APC tumor suppressor (*adenomatous polyposis coli*) in the majority of colorectal cancers [10]. Therefore, target genes of the WNT-pathway should be found to be up-regulated in transcriptome studies. Moreover, specific gene expression changes associated with good or bad prognosis should occur in most studies, as a result of the similar biology that underlies the processes of tumor progression and metastasis. To our great surprise, this was not the case. In fact, the overlap between published gene expression signatures with prognostic relevance for colorectal cancer was very small. This raises questions about the genetic and epigenetic heterogeneity inherent to colorectal cancer, and about the usefulness and stringency of new candidate biomarkers. This review addresses the question whether the current literature reflects the application of gene expression profiling for individualized treatment decisions. In other words, if a clinical practitioner in gastrointestinal oncology or surgery would actually base a therapy decision on the results of a gene expression test for a given patient. Taken together, our aim was to analyze and review reported gene signatures obtained for human colorectal cancer from the viewpoints of tumor biology and clinical practice.

## 2. Results and Discussion

### 2.1. Overview-Proposed Prognostic Gene-Signatures for Early Stage Colorectal Cancer

The focus of our analysis was to investigate biological features related to patient prognosis commonly observed in previous transcriptome studies. Therefore, we reviewed publications between January 2001 and August 2010, in which patient samples from early stage colorectal cancer (UICC stage I-III) were examined by microarray-based gene expression analysis. For that purpose, the PubMed database (http://www.ncbi.nlm.nih.gov/pubmed/) was searched with the keywords ‘gene expression, array, or profiling’, and ‘cancer, tumor, carcinoma, or adenocarcinoma’, and ‘colon, rectal, or colorectal’, and 85 publications were thus selected for this review. In addition, three reports focusing on prognostic markers of colorectal cancer which were previously reviewed by Nannini *et al.* [11] were included in the list. Among the 88 publications, ten are review articles, three are meta-analysis reports and 75 are original research articles (see Table 1). Among the latter 75, the CGH (complete genomic hybridization) or “Exon array” method (Genomic analysis) was used in 12 cases, mRNA-based approaches (cDNA or oligonucleotide microarrays, serial analysis of gene expression / SAGE, cDNA macroarray and low density arrays / LDA) were performed in the remaining 63 studies. In the present review, we focused on original studies on colorectal cancer investigating the mRNA based gene-expression signature with a special emphasis on the prognosis, and not on the expression differences between various cancer stages, or between carcinoma and normal tissue. In 15 articles, candidate mRNA expression signatures were proposed to predict colorectal cancer recurrence (Table 2). The respective study designs, such as the chosen platform of microarray technology, the tumor stage of the patients, and the number of genes of the identified prognostic signature for the recurrence prediction, are shown in Table 2. Since the technical background (e.g., type of microarray technology) and the analytical strategies (as discussed below) vary greatly, a stringent meta-analysis was not appropriate. Instead, we focused on the extraction and identification of the individual genes that were consistently reported in the original studies, according to our hypothesis of common conserved “driver” mutations. In 12 of the 15 studies, prognostic gene expression signatures were identified based on colorectal tumor samples, whereas Barrier *et al.* identified signatures based on the analysis of non-neoplastic mucosa tissue obtained from patients with colorectal cancer CRC (three studies in total). Actually, less than a total of 50 individual gene transcripts were selected as prognostic signature in eight of the studies. To investigate if biological features were consistently observed in these eight studies (*i.e.*, the driver mutations leading to conserved changes in gene transcription), the gene expression signatures were analyzed for overlaps. When the eight independent sets of gene expression were compared, only one gene (APOC1) was identified in more than two independent studies (study No.10 and No.13, Table 2). Interestingly, the other six prognostic expression signatures did not contain the gene APOC1. This unexpected result reveals surprisingly low consistency among the prognostic gene signatures from different studies. The APOC1 gene maps to chromosome 19, it encodes the gene product apolipoprotein C-I which is mainly expressed in the liver, but also has been reported as potential serum biomarker on in colorectal, breast and pancreatic cancer [12-14]. Apolipoprotein C-I has anti-apoptotic and proliferation-enhancing effects on pancreatic cancer cells [15].

### 2.2. Inconsistencies in Gene Signatures between Validated Studies

Even though the prognostic gene expression signatures analyzed here varied greatly and showed no major consistency among them, this does not imply that all prognostic signatures are unreliable. Actually, some of the signatures have been successfully validated. Wang *et al.* performed a microarray analysis using samples from n = 74 UICC II patients, and proposed a candidate 23-gene signature that was associated with disease recurrence [16]. Independently, Barrier *et al.* evaluated this 23-gene expression signature for n = 50 patients with stage II (UICC) colorectal cancer. Importantly, it was reported that the 23-gene signature led to a fairly accurate prediction for the prognosis (overall mean accuracy of 67.1%) [17]. To the best of our knowledge, this was the first report for a prognostic gene signature for colorectal cancer which was successfully validated by an independent research group. Interestingly, Barrier *et al.* developed their own prognostic gene expression signature with 30 completely independent genes using exactly the same patient cohort. This suggests the possibility to develop more than one valid gene expression signature for the prognosis of patients with early stage colorectal cancer. Colorectal cancer is thought to develop over years and decades from early precursor lesions, accompanied by a plethora of epigenetic and genetic alterations. These alterations may not only vary between individual patients, but even within one particular cancer, and thus contribute to expression heterogeneity in colorectal cancer. In addition to this inherent biological “noise”, one has to deal in the clinical context with technical variation, population differences, and the requirement of excellent mRNA conservation in a clinically resected tumor specimen.

However, the central hallmarks of cancer, as defined by Weinberg and Hanahan, require certain pathways to be altered, otherwise the tumor will fail to grow and progress [18]. These changes are certainly linked to expression differences, and it is therefore likely that, even though heterogeneous gene expression renders the task difficult, valuable prognostic information can be derived from transcriptome data sets. A large, yet limited number of completely different prognostic “gene expression signatures” may exist that allow the stratification of patients.

In accordance, our group has obtained similar observations [19,20]. In a cooperation between surgical centers in New Zealand and Germany, n = 147 patients from New Zealand (UICC stage I-IV), and n = 55 German patients (UICC stage I and II) were investigated. Two independent platforms were applied, using oligonucleotide printed microarrays for the New Zealand samples and Affymetrix microarrays for the samples from Germany. This approach yielded a 22-gene prognostic expression set from the New Zealand cohort, and a 19-gene expression set from the German cohort. Importantly, in spite of the differences in the technology and in the clinical background of the cohorts, both prognostic candidate gene signatures retained prognostic power when applied to the alternate series of patients, in a so-called “cross-validation” approach. However, similar to the findings discussed above, both “prognostic expression signatures” were mutually exclusive and contained just one overlapping transcript, the gene TOPK (T-LAK cell-originated protein kinase, or PDZ-binding kinase). The gene TOPK has been assigned to chromosome 8, it encodes a protein kinase which is mainly expressed in testis and cells of the lymphoid lineage [21]. It has recently been shown that the TOPK kinase plays a role during mitosis in the DNA damage response, and directly interacts on the protein level with the tumor suppressor p53 [22]. Thus, it seems evident that there is to date no unique gene expression signature that would represent the best or “exclusive” set of genes for predicting disease recurrence, even based on independently cross-validated studies. Rather, it could be expected that a comprehensive approach that takes multiple independent gene expression signatures into account may yield a robust prognostic tool with the sensitivity and specificity levels required for actual clinical needs.

### 2.3. Strategies for the Development of Clinically Useful Prognostic Gene Signatures

The surprisingly low consistency between independently published prognostic gene expression signatures may be caused by different set-ups in the strategies of the studies. Two quite different approaches are usually applied to place patients into two groups, in order to develop prognostic gene expression signatures with the ultimate goal of distinguishing patients with good and poor prognosis.

The first approach is to investigate the putative molecular nature of eventual metastasis based on the primary tumor itself. Since tumor-related death is in most cases attributable to metastasis, as mentioned by Ramaswamy *et al.* [23], patients with “metastasis-like” gene expression signatures in the primary tumor are likely to have a significantly worse clinical outcome. As stated earlier, metastatic spread in colorectal cancer is observed frequently in the liver, even though many other sites like the lung, brain and peritoneal cavity can be affected as well. The local microenvironment of these different compartments varies greatly in terms of extracellular matrix composition and local growth factor production. In the case of breast cancer, it has been proposed that glial cells produce cytokines which contribute in a paracrine way to the formation of brain metastasis [24]. However, it is still unclear which molecular mechanisms determine the site of distant metastasis in colon cancer. Ramaswamy *et al.* identified the expression profiles of 17 genes that distinguish primary and metastatic adenocarcinoma, based on the analysis of metastatic and primary tumor tissue of diverse origin, such as lung, breast, prostate, large intestine, uterus and ovary [23]. Indeed, tumors bearing a “metastasis-like” gene-expression signature at diagnosis were more likely to develop metachronous distant metastases. However, it should be noticed here that a considerable proportion of the refined gene-expression signature associated with metastasis seems to be derived from non-epithelial stroma components of the tumor. Therefore, RNA extraction from microdissected samples as opposed to non-dissected tumor samples, which may contain considerable stroma components, may also contribute to heterogeneity.

In a similar approach, Yamasaki and co-workers analyzed gene expression profiles of n = 104 samples corresponding to the oncogenic development in an assumed chronological order, including normal mucosa, locally restricted and metastasized primary tumors, and liver metastasis [24]. Transcripts were classified into four distinct groups: (A) genes differentially expressed during all tumor stages, (B) genes differentially expressed in synchronous or metachronous metastasized primary tumors and liver metastasis, (C) genes differentially expressed specifically in liver metastasis, and (D) genes that were not characterized by recognizable expression patterns at all steps of cancer development [24]. It was found that a set of 119 genes of type B allowed the classification of tumors into two classes, the “localized” and the “metastasized” class. Importantly, the disease-free survival and overall survival were significantly longer in the “localized” class than the “metastasized” class [24].

The second approach is to directly compare differential gene expression between patients from similar tumor stages but with good or poor prognosis. Jorissen and co-workers directly focused on the clinical tumor stage according to Dukes' staging system, and were able to determine 128 genes which showed reproducible expression change between Dukes stage A and D cancer [25]. Using this gene signature, the intermediate-stage cancers (stage B and C) were classified into “stage A-like/good prognosis” or “stage D-like/poor prognosis” subtypes [25]. The treatment-adjusted hazard ratio for the recurrence in “stage D-like” cancers was 10.3 for stage B patients, and 2.9 for stage C patients, respectively. Thus, metastasis-associated gene expression changes can be used to refine the traditional outcome prediction which is mainly based on the histopathological TNM-staging system.

Finally, differences in statistical analysis methods may yet be another possible reason for the low consistency between gene signatures from different groups. Cavalieri *et al.* compared three frequently used statistical methods: the significant analysis of microarray (SAM) tool, the Trend Filter tool, and Cox's proportional Hazard Model [26]. SAM is specified as a “one-class-method”, which tests the hypothesis of over- or under-expression for every gene relative to the reference gene, controlling for the false discovery rate (FDR). The Trend Filter tool was derived from the Trend Capture feature of the commercial package “Rosetta Resolver” developed by Rosetta Biosoftware. In the routine Trend Filter tool, a gene is said to exhibit a “trend” in an experimental group if it is over- or under-expressed in a set proportion of the patients in the group. Conversely, a gene does not exhibit a “trend”, if its over- or under-expression is limited to a small proportion of the patients in the group. Finally, the widely used Cox's Hazard model assumes that the underlying hazard rate is a function of several independent variables. In the model used by Cavalieri and co-workers, the parameters age, sex, Dukes' stage and tumor localization were considered as covariates, and one regression coefficient for each gene was obtained [26]. Next, a probabilistic clustering algorithm was performed, and the genes grouped in the more extreme clusters were considered. With this approach, two groups of genes were identified grouped in the highest and in the lowest clusters. As a result, seven genes were retained as prognostic markers by both SAM and Cox Hazard analysis, and only a single gene was commonly retained by the Cox Hazard model, and the Trend Filter analysis [26]. Thus, different statistical methodology may contribute to greatly varied gene expression signatures.

### 2.3. Heterogeneity in Colorectal Cancer Tissue—Stroma Contributions

Great interest has been shown in the biological characterization of the identified genes from the various individual signatures. For example, activity variations of metabolic pathways could be identified through the study of the expression of genes attributed to these pathways in public database (KEGG, Reactome, GenMAPP). A comprehensive pathway analysis showed that patients with favorable prognosis had several activated metabolic pathways, including carbon metabolism, transcription, amino acid and nitrogen metabolism, signaling and fibroblast growth factor receptor pathways. [26]. Moreover, the cell-biological heterogeneity of colorectal cancer is another crucial aspect to be considered for prognostic gene signatures, especially for studies that originate from non-microdissected tumor specimens. We could show that a significant part of the prognostic gene expression signature in colon cancer concerned genes involved in immune modulation, such as chemotaxis-inducing cytokines which regulate the tumor stroma interaction, e.g., by attracting T-cells or modulating angiogenesis [19,27]. There is some evidence that interactions between stromal and cancer cells are a prerequisite for metastases to occur [28,29]. Though it remains unclear whether this metastatic potential originates in cancer cells and/or in stroma compartments, it may be present from the start of the tumor [23,30]. Accepting this theory, the non-neoplastic, normally appearing mucosa on which the tumor has arisen may contain some helpful information. Barrier *et al.* compared gene expression profiles of tumor samples and adjacent non-neoplastic colon mucosa. Three datasets were generated, including the gene expression ratios in tumor samples (T), in adjacent non-neoplastic mucosa samples (A), and the log-ratio of the gene expression measures (L) [31]. Unexpectedly, A-based predictors were more stable (*i.e.*, less sensitive to changes of parameters, such as number of genes and neighbors) than T- or L-based predictors, suggesting the potential usefulness of the non-neoplastic mucosa in predicting the prognosis of colon cancer patients with stage II and III tumors. In addition, the same group further assessed the possibility of developing a prognostic gene signature [17]. Adjacent non-neoplastic colon mucosa samples were collected at a distance between 5 and 10 cm from the gross tumor limit at the time of surgery, and a 70-gene expression signature was developed based on microarray gene expression measures. Interestingly, in contrast to tumor-based gene signature, the non-neoplastic mucosa-based gene signature was suggested to be more specific than sensitive, that is, more able to detect patients with a good prognosis.

Thus, the heterogeneity in colorectal cancer undoubtedly arises in part by varying stroma contributions. However, genetic heterogeneity between the cancer cells themselves may additionally increase the difficulty in developing prognostic gene signature. Baisse and co-workers reported an intratumoral genetic heterogeneity for advanced sporadic colorectal carcinoma [32]. It has been found that more than 60% of the analyzed tumors were heterogeneous for at least one genetic alteration. Actually, intratumoral heterogeneity was more frequently observed in the form of LOH (loss of heterozygosity) than in the form of point mutations. Notably, 67% and 58% of LOH events were heterogeneous at the APC (*adenomatous polyposis coli*) and DCC (*deleted in colon cancer*) tumor suppressor gene loci, respectively, and 20% of point mutation of either the KRAS or TP53 gene were observed to be heterogeneous. Consistent with these findings, it has been pointed out that there are higher “noise” levels in the expression data from colorectal cancer than in those of other solid cancers [33]. To perform a more reliable expression profiling analysis, Yoshida *et al.* combined the analysis of DNA copy number aberration with a comprehensive gene expression screening using n = 79 cases of colorectal cancer (UICC stages I to IV) [33]. Since chromosomal aberrations and genomic instability are often observed in sporadic colorectal cancer, this impressive effort seems well justified. This approach led to the identification of four genes associated with tumor recurrence, four further genes associated with node status and one gene associated with distant metastasis. High expression level and copy number gain of the S100 calcium binding protein A2 gene (S100A2), abhydrolase domain containing 2 (ABHD2), as well as low expression level and copy number losses of ABHD12 (abhydrolase domain containing 12), and the oncoprotein induced transcript 3 (OIT3) were found in recurrent cases compared with non-recurrent cases [33]. This combined analysis of copy-number analysis and gene expression profiling could be helpful in understanding the clinic-pathological meaning of genomic instability.

## 3. Conclusions

Microarray-based classifiers for cancer prognosis have become statistically highly reliable, as mentioned by Fan *et al.* [34]. Several prognostic gene expression signatures have been successfully developed so far for colorectal cancer, and independent validation could be obtained in some cases. However, there is surprisingly little overlap in the gene expression signatures published so far. The low consistency between the different studies may be in part attributed to methodological and technical variances, but it seems evident that colorectal cancer is an extremely heterogeneous disease at the molecular level. This precludes a wide-spread use of expression profiling for clinical purposes, and a selection of an ideal and personalized therapy regime based on gene expression signatures remains elusive. Therefore, it seems evident that further multicenter study and standardized meta-analysis are required. Moreover, currently developed next-generation sequencing approaches may provide additional prognostic information which may not be obtained only by transcriptome profiling. Nevertheless, from a biological point of view, the reported gene expression signatures have largely contributed to our current understanding of tumor initiation and progression in the large intestine.

## Figures and Tables

**Table 1.** Overview: selected literature on microarrays.

Articles Examined		88

	Reviews	10
	Meta-analysis	3
	Research article	75
Research article		

	Genomic analysis	12
	mRNA expression profile	63

**Table 2.** Literature on microarrays - sorted by approach, number of cases, and tissue type analyzed.

**No**	**First Author**	**Year**	**Platform**	**Tumor Stages/Patient Cohort Analyzed**	**Number of Genes in the Prognostic Expression Signature**	**Tissue**
1	Yoshida	2010	oligonucleotide	Stage I-IV CRC	4 genes	tumor
2	Jorissen	2009	oligonucleotide	Dukes' B/C CRC	128 genes	tumor
3	Cavalieri	2007	cDNA	CRC of all stage	8 genes	tumor
4	Lin	2007	oligonucleotide	CRC of all stages (NZ) / Stage I/II CRC (GE)	22 genes (NZ) / 19 genes (GE)	tumor
5	Yamasaki	2007	cDNA	Stage II/III CRC	119 genes	tumor
6	Barrier	2007	oligonucleotide	Stage II CC	70 genes (NM-based)	NM
7	Bianchini [35]	2006	cDNA	Stage I-III CRC (Dukes' B/C)	88 genes	tumor
8	Barrier	2006	oligonucleotide	Stage II CC	30 genes	tumor
9	Arango [36].	2005	oligonucleotide	Dukes' C CC	5-nearest neighbors and 17 genes	tumor
10	Eschrich [37].	2005	cDNA	Dukes' stage B-D CRC	43 genes	tumor
11	Barrier	2005	oligonucleotide	Stage II/III CC	47 genes (A-based)	NM
12	Barrier	2005	oligonucleotide	Stage II/III CC	30 genes (T-based), 70 genes (NM-based)	tumor,N M
13	Wang	2004	oligonucleotide	Dukes' B CC	23 genes	tumor
14	Bertucci [38].	2004	cDNA	Stage I-III CRC	244 cDNA clones	tumor
15	Ramaswamy	2003	oligonucleotide	lung, breast, prostate, colorectal, uterus, ovary	17 genes	Tumor

## Abbreviations

T-based: tumor based
NM-based: non-neoplastic mucosa based
A-based: adjacent non-neoplastic mucosa based gene signature
NM: non-neoplastic mucosa
CRC: colorectal cancer
CC: colon cancer
